# Treatment-Related Reversible Cerebral Vasoconstriction Syndrome

**DOI:** 10.3390/jcm13195930

**Published:** 2024-10-04

**Authors:** Giulia Avola, Alessandro Pezzini

**Affiliations:** 1 Department of Medicine and Surgery, University of Parma, 43121 Parma, Italy; 2 Stroke Care Program, Department of Emergencies, Parma University Hospital, 43126 Parma, Italy

**Keywords:** reversible cerebral vasoconstriction syndrome (RCVS), thunderclap headache, reversible vasospasm, treatment-related RCVS, endothelial dysfunction, dysregulation of cerebral vascular tone, sympathetic over-reactivity, convexity subarachnoid hemorrhage (cSAH), sausage on strings

## Abstract

Reversible cerebral vasoconstriction syndrome (RCVS) is a rare but significant cause of intracranial arteriopathy and stroke in young adults. The syndrome encompasses a spectrum of disorders radiologically characterized by reversible narrowing and dilation of intracranial arteries, often triggered by vasoactive drugs or the postpartum period. The hallmark clinical feature of RCVS is thunderclap headache with or without other neurological signs. Though endothelial dysfunction and sympathetic hyperactivation are hypothesized to be key mechanisms, the exact pathogenesis of RCVS is still unclear. RCVS’s diagnosis could be challenging, since vasospasm proceeds centripetally, initially involving distal small pial and cortical arteries, and angiographic studies, especially brain magnetic resonance angiography (MRA) and computed tomography angiography (CTA), may miss it in the early phase of the disease, while early signs such as vascular hyperintensities may be visible on T2/FLAIR sequences before vasospasm onset. Catheter angiography is the gold standard and it could be used to assess vasospasm reversibility post-intra-arterial vasodilator administration. Treatment is mainly symptomatic, and nimodipine is the most commonly administered therapy, given orally or intra-arterially in severe cases. Since many aspects of RCVS remain partially known, further research is needed to better understand the complex pathophysiology of this unique clinical condition and to optimize specific management strategies.

## 1. Introduction

Reversible cerebral vasoconstriction syndrome (RCVS) is a clinical–radiological entity whose main characteristic is the reversible constriction of segments of intracranial arterial vessels. Although a group of pathologies characterized by reversible narrowing and dilation of cerebral arteries has been reported since the early eighties, the disease has been consistently recognized only in the last two decades, and the unifying term RCVS was proposed in 2007 [1], overcoming the previous eponyms “Call Fleming syndrome”, “thunderclap headache with reversible vasospasm”, “benign angiopathy of the central nervous system”, “postpartum angiopathy”, “migrainous vasospasm or migraine angiitis” and “drug-induced cerebral arteritis or angiopathy” [2,3,4,5,6,7]. The pathogenic mechanism underlying RCVS is still unclear. However, growing evidence indicates that endothelial dysfunction and sympathetic hyperactivation play a role and that specific drug-related mechanisms can lead to disease occurrence in many cases [8,9,10].

In the present review article, we will summarize the most recent advances in this relatively new disease entity, focus on its variable phenotypes and radiological features, and pose additional questions on the biologic process leading to cerebral vasoconstriction, with an emphasis on drug-related mechanisms.

## 2. Epidemiology

The estimated incidence of RCVS is about three per million in adults [11], according to the number of presenting patients to the Emergency Department, but it is likely higher. However, RCVS is one of the most common forms of intracranial arteriopathy and a cause of stroke in young adults [12]. Although the disease has been reported to affect any age group, the average age of occurrence is 40–55 years, with a prevalence in females [2]. Male patients tend to be younger (fourth–fifth decade in women vs. third–fourth decade in men) [13] or adolescents (>85% of affected adolescents are male) [14], but the reason for this disparity is unknown. Additionally, male sex is more often associated with a better outcome [9].

## 3. Clinical Presentation

A total of 85–100% of cases present at onset with thunderclap headache, which is described as the worst headache in life, with sudden onset, reaching its maximum intensity in ≤1 min and often accompanied by crying, photophobia, nausea and vomiting, but also seizures and hypertensive crises during the attack [15,16].

The headache episodes are often triggered by coughing [17], bathing [18], physical exertion, sexual activity [19], the Valsalva maneuver and emotional outbursts [2]. Headaches are recurrent and persist for up to four weeks; they can arise two to three weeks before the overt clinical manifestations of RCVS, which are characterized by the association of other focal neurological manifestations with headache [20]. Between episodes, a form of mild to moderate intensity holocranial headache persists [12]. Neurological signs (present in 8–43% of cases) depend on RCVS complications, including cerebral ischemia, cerebral hemorrhage and posterior reversible encephalopathy syndrome (PRES) [9,21].

PRES and RCVS share some clinical and radiological features, and they can occur in the same patient. PRES is more commonly characterized by impaired visual acuity or visual field deficits, disorders of consciousness with seizure and encephalopathy, and ischemic stroke or intracerebral hemorrhage in more severe cases [22]. Whether PRES and RCVS are independent syndromes or a part of a continuum process is still debated; in both syndromes, blood flow dysregulation along with endothelial dysfunction and blood–brain barrier breakdown are the main pathophysiological mechanisms [22].

Depending on the affected area, aphasia, hemiparesis, hemianopia, seizures or Balint’s syndrome can be observed [21]. The incidence of seizures, however, is rare (7–17% of cases) [9]. Table 1 shows the diagnostic criteria of headaches attributed to reversible cerebral vasoconstriction syndrome, according to the Headache Classification Committee of the International Headache Society (IHS) [20].

## 4. Pathogenesis

RCVS can occur spontaneously (primary RCVS), in the absence of a predisposing factor, but in 40–60% of cases, it is induced by factors considered triggers (secondary RCVS) [2]. Spontaneous RCVS predominates in Asian populations, while trigger-induced RCVS prevails among Europeans and Americans [2,23]. Although the exact pathophysiological mechanisms underlying vasoconstriction are still unknown, the following two main pathophysiological hypotheses have been put forward in the last decade: (1) alteration of vascular tone and (2) endothelial dysfunction.

### 4.1. Dysregulation of Cerebral Vascular Tone and Sympathetic Over-Reactivity

The pial cerebral vessels receive rich innervation from the superior cervical ganglion, the sphenopalatine ganglion and the trigeminal nerve, whose nerve endings release vasoactive substances such as neuropeptide Y and norepinephrine, resulting in vasoconstriction [24]. Similarly, cerebral microcirculation receives sympathetic innervation from the locus coeruleus, the raphe nucleus and the basal forebrain [24]. Vascular tone regulation is made possible by different groups of receptors. Norepinephrine exerts vasoconstriction of the middle cerebral artery (MCA) through α-1 adrenergic receptors [25] and vasodilation of parenchymal arterioles through β-adrenergic receptors [26]. An imbalance in vascular tone, driven by increased sympathetic activity on the vessel walls, causes the characteristic vasoconstriction seen in RCVS. Studies on cerebrovascular vasoreactivity support this hypothesis. Specifically, a retrospective case series revealed significant impairment of the breath holding index during the acute phases of the disease, reflecting compromised vasodilatory capacity in response to hypercapnia [27]. In support of this hypothesis, recent studies have suggested a genetic predisposition to the dysregulation of the vascular tone through the identification of circulating microRNAs that target genes responsible for vascular tone, such as the gene encoding for endothelin 1, or those involved in the transforming growth factor beta (TGFβ) signaling pathway in RCVS patients [28]. A polymorphism (val66Met) in the brain-derived neurotrophic factor (BDNF) gene has also been identified, which might be responsible for the severity of vasoconstriction in RCVS [29], as it upregulates neuropeptide Y. On the other hand, an aberrant response to sympathetic stimulation can make patients more vulnerable to vasoconstriction [9]; this could explain why some patients develop RCVS after the intake of sympathomimetic agents.

### 4.2. Endothelial Dysfunction

As endothelial cells secrete vasoconstrictive and vasodilatory substances acting on the muscular component of the arterioles wall, they contribute to the maintenance of cerebral blood flow through mechanisms of vasoconstriction and vasodilation. The integrity of the endothelium is, therefore, crucial in this regard. It has been demonstrated that patients with RCVS also have fewer CD34KDR endothelial progenitor cells compared to controls, especially those with more severe vasoconstriction [30]. Since these cells are responsible for re-endothelialization [31], it is possible that in the acute phases of the disease, patients who have suffered endothelial damage also have a deficit in the mechanisms of endothelial repair. Endothelial damage could be favored by sympathetic hypertonia (or hyper-reactivity to sympathetic stimulation) or by excessive oxidative stress [2]. In fact, in the acute phases of the disease, increased levels of 8-iso prostaglandin F2α have been shown in both urine and plasma [32]. The 8-iso prostaglandin F2α is considered a marker of oxidative stress and has a strong vasoconstrictive effect. The result is the breakdown of the blood–brain barrier (BBB), leading to increased permeability of the vascular wall, which is radiologically observed with contrast-enhanced FLAIR in 50% of cases [33]. Recently, a technique called dynamic contrast-enhanced magnetic resonance imaging (DCE-MRI) has demonstrated increased microscopic permeability in the acute phase, indicating that barrier damage is present even when not microscopically visible [34]. Finally, a study based on high-resolution vascular wall imaging has shown enhancement of the affected vessels in almost half of the patients with RCVS, suggesting that a perivascular inflammatory process might also contribute to endothelial damage [35]. Although these mechanisms are of great biological interest, evidence supporting this hypothesis is scarce and controversial. The difficulty in obtaining histopathological samples of the affected vessels is just one of the major limitations in understanding the mechanisms regulating cerebral vasoconstriction [2].

### 4.3. Pathogenesis of Thunderclap Headache

The pathophysiological mechanism underlying thunderclap headache is unclear, but it is unlikely to depend on the vasoconstriction of large vessels because vasoconstriction occurs approximately two weeks after headache onset. More likely, the involvement of distal cerebral and pain-sensitive pial vessels leads to the activation of the trigeminovascular nociceptive pathway [36], resulting in the release of vasodilatory substances such as calcitonin gene-related peptide (CGRP). Physiologically, the trigeminovascular reflex restores vascular tone after a vasoconstrictive stimulus [15].

Furthermore, cerebral blood vessels are innervated both by sensory afferents from the trigeminal nerve and the dorsal root of C2; this could be the reason why the headache is linked to the involvement of cerebral vessels [37].

Trigeminal nociceptors could also be activated by leaked intravascular components when BBB permeability is altered [9]. In patients with RCVS, these mechanisms seem to be altered.

### 4.4. Trigger Factors in RCVS

The same factors that can trigger thunderclap headache are also precipitating factors for RCVS, where intense headache is associated with other focal neurological signs and neuroradiological findings. However, the two most common triggering conditions (approximately 31% of cases) are the intake of vasoactive drugs and the postpartum period [2,15,38]. The pathogenic mechanism might be slightly different in these two subcategories. Table 2 summarizes the potential precipitating factors and hypothetical triggers for RCVS [9].

Vasoactive drugs act through specific receptors located in the smooth muscle and cause overstimulation of the sympathetic system on the vessel wall. In 1962, a study involving the direct injection of serotonin into the carotid arteries of monkeys showed 50% narrowing of the internal carotid artery and a consequent significant increase in cerebral perfusion pressure [39], as an initial autoregulation mechanism to maintain a constant cerebral perfusion flow (CBF). This explains the association of RCVS with the intake of selective serotonin reuptake inhibitors (SSRIs) and serotonin–norepinephrine reuptake inhibitors (SNRIs). Other case reports suggested that drugs that act as agonists of serotonergic receptors, such as triptans [40,41] and benzodiazepines (e.g., clonazepam), can cause vasoconstriction of cerebral arteries through the activation of serotonin receptors, thus precipitating RCVS [42]. For the same reason, some authors have hypothesized a possible association with sympathomimetic agents that have serotonergic effects [9].

Another class of drugs associated with RCVS is immunosuppressants. Several case reports, for example, have focused on the potential effect of Fingolimod, an agent approved for the treatment of multiple sclerosis. Fingolimod acts as an inhibitor of the sphingosine 1-phosphate receptor, thus inhibiting lymphocyte migration to sites of inflammation; additionally, since sphingolipids have a vasoactive effect, their inhibition can explain vasospasm. Specifically, the inhibition of ceramide, which is a vasodilator, causes vasoconstriction [43]. During the phase three Fingolimod trial, a case of peripheral vasoconstriction was also described in patients taking a higher dose of 1.25 mg daily [44]. Interferon is also a possible trigger for RCVS. Raynaud’s phenomenon and livedo reticularis, both characterized by vasoconstriction of small arteries and peripheral arterioles, have been described as further possible side effects of treatment with interferon [45,46], which supports the hypothesis that interferon may have a vasoactive effect on cerebral vessels as well. Specific anticancer and other immunosuppressive drugs have been reported as possible RCVS triggers in sparse case reports. Among these, methotrexate, an immunosuppressant used in hematological cancers that acts as a dihydrofolate reductase inhibitor, was shown to exert neurotoxic effects, including vasoconstriction, primarily at high doses, typically after intravenous administration of at least 1000 mg/m² or after intrathecal administration [47]. The triggering effect of anti-CGRP monoclonal antibodies, in this regard, is actually unproven as it is based on only a few case reports and the patients had also taken triptans for headache at the same time [48]. CGRP is a potent vasodilator that acts on the cardiovascular and cerebrovascular systems, so its sustained inhibition could theoretically favor cerebral vasospasm [49]. However, it is unclear whether the administration of the antibody alone is sufficient to trigger RCVS. Additionally, CGRP receptors are located in the smooth muscle, so the luminal administration of these antibodies does not allow them to reach muscle cells and trigger vasospasm unless there is barrier disruption [50]. Therefore, the monoclonal antibody alone, in the absence of pre-existing barrier damage, might not be sufficient to induce RCVS.

The association of RCVS with other immunosuppressive treatments (steroids, mycophenolate mofetil, hydroxychloroquine) also explains the association of RCVS with conditions such as systemic lupus erythematosus (SLE), antiphospholipid syndrome (APS) and systemic sclerosis (SS) [51,52]. In these cases, RCVS is not only treatment-related, as endothelial damage is also caused by the diseases themselves. In particular, in APS, autoantibodies affect the coagulation cascade and activate endothelial cells that, in turn, release vasoactive agents such as endothelin 1 (ET1) [53]. ET1 regulates the intracellular influx of calcium and thus modulates the muscular tone of the vessel. Indeed, a decrease in plasma ET1 levels has been demonstrated after the resolution of vasospasm in patients with RCVS [52]. Another noteworthy discovery is that cilostazol, by inhibiting ET1’s effect on vascular walls, may potentially alleviate vasospasm, making it a reasonable treatment option for RCVS associated with APS [54].

The role of immunosuppressants as potential triggers also explains the association of RCVS with transplantation. In particular, tacrolimus has been hypothesized as the leading cause of transplantation-related RCVS [55]. In this case, the neurological symptoms caused by RCVS appear within two weeks of the transplant and could be irreversible due to the neurotoxicity of the drug. Therefore, the administration of tacrolimus should be gradual and closely monitored with plasma level measurements. It has also been observed that its discontinuation, rather than dosage reduction, reduces long-term neurological sequelae.

Other possible triggers identified in the literature include ergot derivatives, erythropoietin, intravenous immunoglobulins, anesthetic drugs and antimuscarinic drugs. In particular, some case reports suggested that RCVS may be potentially related to oxybutynin therapy. This antimuscarinic drug acts selectively on the M3 receptor, which is one of the most expressed receptors in the brain parenchyma, as well as M1 and M5 receptors. When M3 receptors are bound, they mediate vasoconstriction through the activation of pericytes or vasodilation through the release of endothelial nitric oxide. As a result, by blocking M3, oxybutynin could modify the balance between vasoconstriction and vasodilation [56].

RCVS can also occur after a red blood cell transfusion. The brain is not commonly exposed to blood transfusion reactions. However, it has been hypothesized that the autoregulatory response of vessels to changes in blood volume and oxygenation could be the main pathogenic mechanism [57]. Chronic anemia leads to hypoxemia and thus vasodilation [58]; a rapid correction of hemoglobin, with an increase in its value ≥ 5 g/dL, causes a loss of vasodilation [59]. Additionally, the increase in hematocrit and thus blood viscosity can cause endothelial damage and hence vasospasm [60]. The timing of these mechanisms varies; cases where RCVS develops immediately after the transfusion and cases where it develops about a week later have been described [57].

Other potential triggers include illicit drugs such as 3,4-methylenedioxymethamphetamine (ecstasy), cocaine, amphetamines, lysergic acid diethylamide (LSD) and tetrahydrocannabinol [61]. Even more commonly used drugs, such as non-steroidal anti-inflammatory drugs (NSAIDs) and contraceptive pills, have been reported as potential triggers. NSAIDs are not commonly associated with RCVS, but a few case reports have highlighted a possible association between the administration of indomethacin and RCVS. The mechanism by which they induce RCVS is not known, but their effect is likely mediated by the inhibition of cyclooxygenases [62]. Indomethacin, in particular, inhibits prostacyclin, which has a vasodilatory effect, and unlike other NSAIDs, it counteracts nitric oxide-induced vasodilation, thereby favoring vasoconstriction [63].

Some case reports also support that RCVS can be triggered by hypercalcemia following calcium supplementation [64]. Animal models have shown that calcium acts in the process of actin–myosin binding within cerebrovascular smooth muscle cells to promote contractility and causes inflammation and endothelial damage, leading to the permeability and breakdown of the blood–brain barrier [65]. The serum calcium value above which this alteration is observed is not known; in several cases, a critical value > 13 mg/dL has been reported [64].

Finally, contraceptive pills may play a role as a trigger factor due to their estrogen content. Estrogens act as modulators of vascular tone and consequently of cerebral blood flow. Specifically, they reduce the effects of the sympathetic system, stimulate the production of nitric oxide synthase by the endothelium and its phosphorylation, promoting vasodilation, and favor the production of prostacyclins, with consequent vasodilatory effects [66]. However, the role of estrogens in the pathogenesis of RCVS remains currently unclear. An association of the disease with contraceptive pills has been proposed [67], as well as with hormonal stimulation for intrauterine insemination [68] and intrauterine devices releasing levonorgestrel [69]. Of note, a sudden reduction in estrogen levels may also favor the onset of RCVS; as mentioned previously, the postpartum period (2 weeks after delivery) is a predisposing condition, more often associated with PRES (>25% of cases), which in 7–38% of cases is associated with RCVS [9]. The abrupt reduction in estrogens in the postpartum period could therefore explain the recurrence of RCVS during this time frame. For the same reasons, treatments such as bilateral salpingo-oophorectomy have been proposed as triggers, since they induce a sudden reduction in estrogen production [70].

However, the major risk factor in postpartum-related RCVS is thought to be eclampsia. The placenta plays a crucial role in the development of eclampsia. The placenta secretes pro-inflammatory cytokines, pro-angiogenic proteins such as placental growth factor and its soluble receptor, and soluble endoglin into the maternal circulation, leading to an immune response and subsequent endothelial dysfunction, which likely causes vasoconstriction [71].

Despite the growing evidence, treatment-related RCVS is mainly described in case reports, so further research is necessary to fully understand the role of drugs or estrogens. Additionally, to make the issue even more complicated, only limited experience suggests that re-exposure to these trigger factors does not lead to relapse. As biologically hard to explain, this implicates that, if clinically indicated, vasoactive drugs can be re-prescribed to the patient.

## 5. Pathophysiological Models

**Figure 1:**
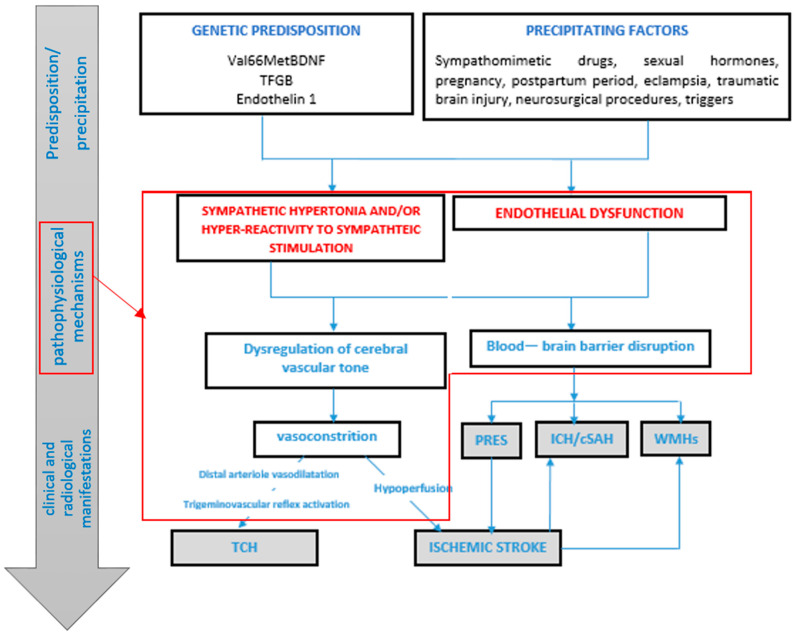
The proposed model of the pathophysiology of RCVS. The development of RCVS is sequential, which may require both predisposition and precipitating factors to initiate and perpetuate a vicious cycle of pathogenic mechanisms (showed in the red box) that result in the clinical and radiological manifestations of RCVS (as indicated by the gradient arrow on the left of the figure). Dysregulation of cerebral vascular tone and disruption of the blood–brain barrier (BBB) are supposed to be crucial in the pathophysiology of RCVS. Both of them are a consequence of endothelial disfunction, sympathetic overactivity and oxidative stress, mediated by mechanical and biochemical stimuli. When the autoregulation and BBB disruption worsen and the endogenous protective mechanisms fail, headache, vasoconstriction and complications may ensue. In particular, hemorrhagic complications (cSAH and ICH) or PRES may be attributed to the breakdown of the BBB, while ischemic stroke is related to hypoperfusion caused by vasoconstriction of major cerebral arteries. White matter hyperintensity lesions could be attributed to either increased BBB permeability or partial ischemia due to cerebral hypoperfusion. The thunderclap headache could be attributed to the dilatation of distal arterioles or meningeal arteries that activate the trigeminovascular nociceptive fibers. RCVS: reversible cerebral vasoconstriction syndrome. PRES: posterior reversible encephalopathy syndrome. ICH: intracerebral hemorrhage. cSAH: convexity subarachnoid hemorrhage. WMHs: white matter hyperintensities. TCH: thunderclap headache.

A pathophysiological model of RCVS is illustrated in Figure 1. In patients with a genetic predisposition and therefore greater vulnerability to triggering factors, the release of vasoactive substances such as neuropeptide Y, endothelin 1 and catecholamines leads to vasoconstriction in cases of overstimulation of the sympathetic system. Arterioles respond with initial vasodilation, partially due to trigeminovascular reflex activation, resulting in perivascular nociceptor activation and thunderclap headache. Conversely, in response to peripheral vasodilation, the larger proximal vessels undergo vasoconstriction. The alteration in vascular tone contributes, alongside blood–brain barrier breakdown, to the onset of the clinical and radiological complications of RCVS.

## 6. Imaging Findings

Imaging findings at onset are often normal. Shortly thereafter, changes in neuroradiological findings are a hallmark of RCVS [12].

### 6.1. Brain Magnetic Resonance Imaging (MRI) and Magnetic Resonance Angiography (MRA)

**Figure 2:**
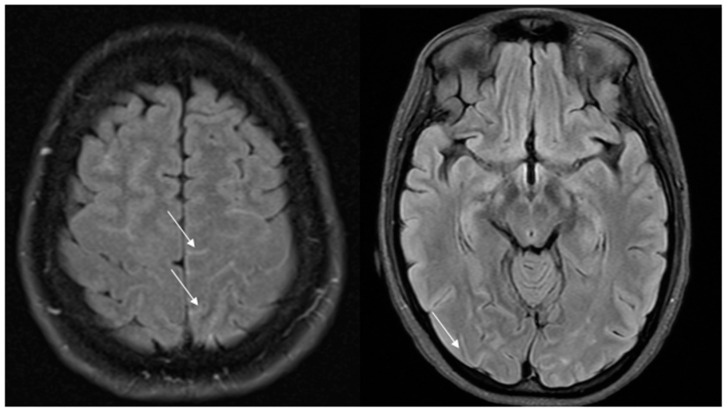
Hyperintense vessels on FLAIR images in two patients with RCVS (arrows).

As previously said, vasospasm initially involves only small distal cerebral vessels not depicted by MRA and then centripetally spreads to larger vessels over days or weeks. As a consequence, initial brain MRI is normal in 30–55% of cases, while initial MRA is normal in 22% of cases [9]. Nevertheless, vascular FLAIR hyperintensities as early signs can be visible on T2/FLAIR sequences, even before vasospasm becomes visible on MRA [72]. Well-circumscribed dot or tubular-like appearances of hyperintense lesions running through the sulci, correlating with slow flow on the cortical surface, have been described. These hyperintense vessels (HVs) are often bilateral, involving the territories of the middle cerebral artery (MCA) and posterior cerebral artery (PCA) (Figure 2).

**Figure 3:**
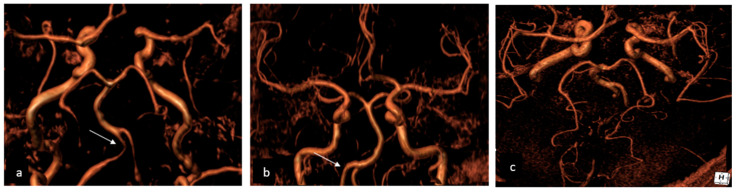
A 58-year-old female admitted to the Emergency Department due to thunderclap headache, diagnosed with RCVS. (**a**) The initial Angio CT showed bilateral V4 vasoconstriction (arrow) and narrowing posterior cerebral arteries; (**b**) three months later, the follow-up Angio CT showed complete resolution of vertebral artery vasoconstriction (arrows); (**c**) the follow-up imaging also showed complete recanalization of posterior cerebral arteries. RCVS: reversible cerebral vasoconstriction syndrome.

More frequently, HVs are observed in patients with PRES and ischemic stroke, suggesting that they may not only serve as a useful marker of RCVS but may also indicate clinical severity. HVs are distinct from subarachnoid hemorrhage, as they do not appear hypointense on SWI sequences [72]. Vasospasm, on the other hand, can be visualized with magnetic resonance angiography (MRA) or through computed tomography angiography (CTA) (as shown on Figure 3) when it starts involving larger cerebral vessels. As a result, the diagnosis could be challenging in the acute phase and often delayed. In cases of treatment-related RCVS, cerebral edema is more frequently observed and vasoconstriction is more widespread and severe compared to the idiopathic form where vasoconstriction is usually segmental.

The addition of contrast medium in T2/FLAIR plus gadolinium sequences is useful as it reflects blood–brain barrier breakdown. It has also been shown that breakdown of the blood–brain barrier can be an independent risk factor for neurological complications such as subarachnoid hemorrhage and PRES [73].

The radiological findings of RCVS are distinct from other vascular pathologies (such as atherosclerosis, dissection, fibromuscular dysplasia, vasculitis). The main mimic is primary central nervous system vasculitis (PACNS). In PACNS, large-caliber vessels of the circle of Willis and their collateral branches are involved. Additionally, vessel wall MRI sequences (vw-MRI) can be useful in the differential diagnosis, since there is no contrast enhancement of the affected arterial wall in RCVS, whereas enhancement is detectable in vasculitis [10,74]. Moreover, while in PACNS only cerebral vessels are involved, involvement of extracranial vessels has been rarely reported in RCVS.

### 6.2. Catheter Angiography

The diagnostic gold standard remains digital subtraction angiography. During angiography, smooth tapered narrowing followed by abnormal dilated segments can be visualized. This alternation of narrowing and dilatations is responsible for the typical “sausage on strings” appearance [75]. Angiography also allows for the exclusion of alternative diagnoses and the study of vasospasm reversibility through intra-arterial administration of vasodilators [36]. This is particularly useful because the diagnosis of RCVS is often made retrospectively once the reversible nature of vasospasm is demonstrated. Being able to assert early in the disease process that vasospasm is reversible significantly advances the diagnosis and enables more informed patient monitoring. In fact, in up to a third of cases, angiographic evaluation is normal within the first week of symptom onset due to centripetally proceeding vasospasm [76].

Finally, normalization of angiographic findings is typically observed within 8–12 weeks. However, the evolution of cerebral vasospasm is not uniform; so, while some vessels improve, others worsen during the monitoring period [76].

### 6.3. Perfusion Imaging

Lastly, there are limited data on the use of perfusion CT; from clinical experience and a few published case reports, we know that perfusion CT shows multiple areas of hypoperfusion, reflecting the effect of vasospasm that can acutely progress to ischemia [77,78]. This information could potentially be used in the future to monitor response to treatment with vasodilators [77].

**Figure 4:**
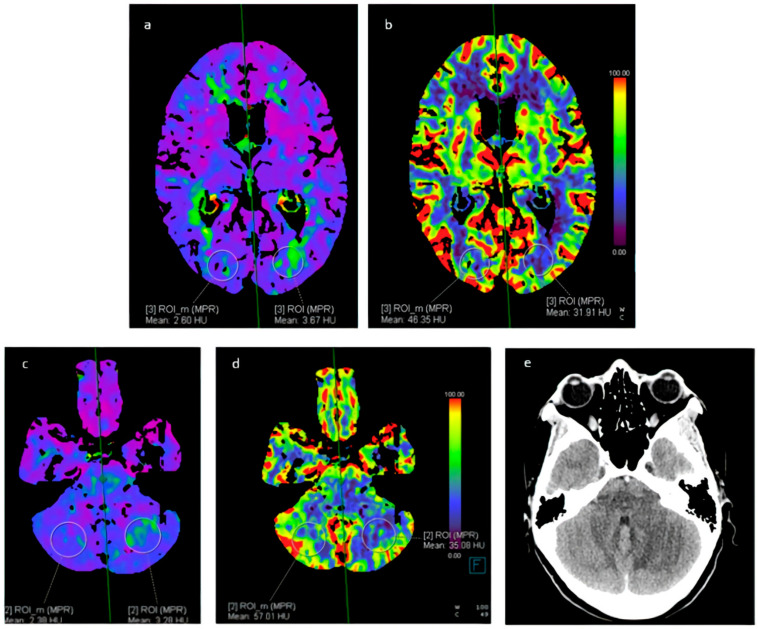
A 37-year-old female with cocaine-induced RCVS, admitted to the Emergency Department due to headache and bilateral hypovision. Perfusion CT showed (**a**,**b**) an increased TMAX and reduced CBF in the left occipital lobe; (**c**,**d**) an increased TMAX and a reduced CBF in the left cerebellar hemisphere. (**e**) CT showed a cerebral infarct on the left cerebellar hemisphere corresponding to the area indicated on perfusion CT. RCVS: reversible cerebral vasoconstriction syndrome. TMAX: time-to-maximum. CBF: cerebral blood flow.

Alternatively, arterial spin-labeling perfusion is a completely non-invasive MRI technique that does not require the administration of gadolinium contrast. Instead, it utilizes electromagnetic spin inversion to label water molecules, which then act as freely diffusible flow tracers [10] (Figure 4).

### 6.4. Transcranial Doppler Sonography

Transcranial Doppler ultrasound can play a role in demonstrating increased morpho-velocimetric profiles suggesting vasospasm and a reduced caliber of internal carotid arteries (ICA), MCAs and anterior cerebral arteries (ACAs) [12,79]. Sonographic studies may also be inconclusive in the early stages, while higher peak velocities are typically observed after approximately 3 weeks. Moreover, peak velocities > 120 cm/s and a Lindegaard index (maximum velocity in MCA/maximum velocity in ICA) > 3 are associated with an increased risk of developing PRES [72]. Patients with hyperintense vessels (HVs) initially exhibit maximum velocities in the middle cerebral artery (MCA) of up to 121.0 ± 39.5 cm/s and a Lindegaard index (LI) up to 2.8 ± 1.2, approaching the cutoff criteria for predicting severe outcomes such as PRES or ischemic stroke (VMCA > 120 cm/s and LI > 3) [72]. This underscores HVs as a severity marker in RCVS. Bedside ultrasound is also useful for follow-up. Normalization of findings in this case is typically achieved within 12 weeks [79].

## 7. Clinical–Radiological Complications

**Figure 5:**
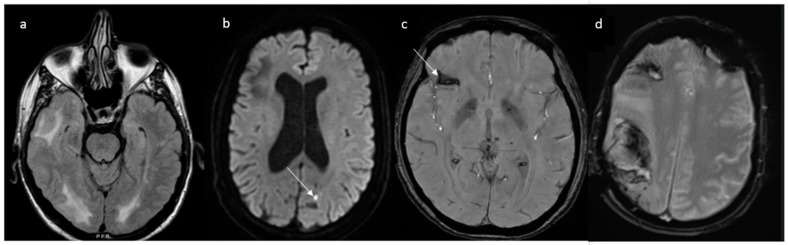
An overview of the potential complications of RCVS in four different patients. (**a**) Posterior reversible encephalopathy syndrome appearing as hyperintense lesions on fluid-attenuated inversion recovery imaging (FLAIR); (**b**) ischemic strokes (arrow) highlighted by diffusion-weighted imaging (DWI); (**c**) convexity subarachnoid hemorrhage shown as the hypointensity, demonstrated by gradient echo imaging that indicates the presence of blood within the cortical sulci; (**d**) temporo-parietal and frontal lobar parenchymal hemorrhages. RCVS: reversible cerebral vasoconstriction syndrome. FLAIR: fluid-attenuated inversion recovery. DWI: diffusion-weighted imaging.

Complications of RCVS are more commonly observed in treatment-related RCVS than in primary RCVS [80]. Up to 81% of patients exhibit the following radiological abnormalities appearing days to weeks after the onset of thunderclap headache: cerebral ischemia (39%), intraparenchymal hemorrhage typically lobar in 20%, convexity subarachnoid hemorrhage in 34% and PRES in 38% [9] (Figure 5).

Cerebral vasoconstriction peaks 9–13 days after the initial thunderclap headache [15,81] and progresses with a distal-to-proximal pattern. This is the reason why ischemic and hemorrhagic complications have a different temporal distribution, occurring at varying times throughout the course of RCVS. Initially, ischemic lesions are due to perfusion deficits caused by altered arteriolar autoregulation and are small, peripheral and cortical (at the cortico-subcortical junction) [9,12]. After approximately two weeks, vasoconstriction of larger-caliber vessels leads to severe hypoperfusion, and the lesions are larger, wedge-shaped and watershed [9]. Vasoconstriction of small distal pial vessels may lead to reperfusion injury with subsequent vessel wall rupture and hemorrhagic stroke typically in watershed areas (as shown in Figure 5), which tends to manifest earlier (2–4 days after the headache attack) than the ischemic counterpart [82]. RCVS is also the leading cause of convexity subarachnoid hemorrhage in individuals aged < 60 years [83]. In these patients, thunderclap headache, FLAIR dot sign and multifocal vasoconstriction are diagnostic of RCVS [12]. A young age, a history of severe headache, chronic obstructive pulmonary disease requiring sympathomimetics (e.g., ephedrine), antidepressant use, a low Hunt Hess score or Fisher score and the involvement of multiple arteries including bilateral are independent predictors of RCVS-SAH [84].

The type of lesion cannot be reliably predicted based on triggering factors, except for eclampsia, since it is associated with both PRES and RCVS.

As previously said, PRES and RCVS share their radiological features, as they are based on the same pathophysiological mechanisms, impaired cerebral regulation, blood–brain barrier disruption and endothelial dysfunction, leading to passive extravasation of fluids and proteins and resulting in vasogenic edema, which appears as hyperintensities on apparent diffusion coefficient (ADC) mapping.

Actually, MRI shows some differences between these two syndromes: In PRES, MRI typically shows symmetric vasogenic edema in the parieto-occipital regions, affecting both the subcortical white matter and the overlying cortex [22]. In contrast, PRES associated with RCVS is more often asymmetric. Additionally, hemorrhages and, less commonly, ischemic lesions have been described in PRES. The primary hemorrhagic pattern in PRES consists of multiple minute foci of microhemorrhages, whereas in RCVS, the most common hemorrhagic patterns are cSAH and ICH [22].

Impaired cerebral autoregulation in cases of PRES is observed in >85% of cases, leading to vasospasm [9]. However, ischemic lesions in PRES are typically small and punctate, and are observed within confluent areas of vasogenic edema, while extensive infarcts are rare [22]. Furthermore, estrogens are supposed to play a role in both pathologies. Female sex, cocaine use and a history of hypertension are likely risk factors for hemorrhagic complications [82].

## 8. Diagnostic Process and Differential Diagnosis

Thunderclap headache, especially if occurring in a single episode, necessitates differential diagnosis with other potentially fatal conditions such as subarachnoid hemorrhage due to aneurysm rupture, parenchymal hemorrhage, posterior or middle cerebral artery embolism, cervical artery dissection, venous sinus thrombosis and brain infections. Useful elements for differential diagnosis include medical history focusing particularly on RCVS triggers, imaging and lumbar puncture [10]. Immediate brain computerized tomography (CT) should be performed and computerized tomography angiography (CTA) should be obtained immediately [12]. If subarachnoid hemorrhage is present, the distribution of blood allows for further evaluations; in cases of RCVS, subarachnoid hemorrhage is most often confined to superficial sulci, which differs from aneurysmal subarachnoid hemorrhage where blood is localized in the basal cisterns/circle of Willis [10]. If brain CT does not show acute findings, lumbar puncture should be performed to rule out subarachnoid hemorrhage and inflammatory conditions [85,86]. Conversely, if lumbar puncture is also normal, brain MRI and MRA are warranted. MRI venography should be considered in order to exclude cerebral venous thrombosis.

PACNS represents the most important mimic due to overlapping characteristics such as headache, stroke and vessel wall irregularities [75]. Distinguishing these two entities is particularly important because glucocorticoids administered in cases of vasculitis could worsen outcomes in patients with RCVS [87]. Table 3 summarizes the main diagnostic findings helpful for differential diagnosis [9]. Patients with primary angiitis of the central nervous system also present with dull chronic headache, scattered deep infarcts and the involvement of smaller cerebral arteries [12]. Additionally, non-contrast CT scanning with multiple ischemic lesions of different ages suggests a form of vasculitis.

Diagnostic criteria have been proposed. The RCVS2 scoring system (Table 4) allows for confirmation of the diagnostic suspicion with high accuracy, excluding mimics such as primary angiitis of the central nervous system [88]. This score ranges from −2 to +10: a score ≥ 5 has 99% specificity for diagnosing RCVS with high sensibility (90%), while a score ≤ 2 has 100% specificity for excluding RCVS (whit 85% sensibility); scores 3 and 4 have moderate specificity (86%) and low sensitivity (10%) for diagnosing RCVS [87].

## 9. Prognosis and Management

Prognosis in RCVS is favorable both in adults and adolescents. More than 90% of patients achieve a good outcome, defined as a Modified Rankin Scale (mRS) score 0–1 [4]. A worse outcome is associated with baseline neurological deficits and the onset of cerebral ischemia in the early phase of the disease [89]. Furthermore, despite the term “reversible”, recurrence can occur in 5–10% of cases [90]. Headache may persist beyond vasospasm resolution; follow-up studies show that about 50% of patients continue to experience chronic headaches for more than a year, and 26% develop depression [91]. In these cases, triptan administration is often avoided due to limited data on the effects of re-exposure to triggering factors. Non-pharmacological approaches should be preferred for antidepressant administration for the same reason [12].

Current treatment recommendations for RCVS are primarily based on large observational studies, smaller case series and expert opinions, as no randomized controlled trials have been conducted [92]. Treatment remains symptomatic to date; once trigger factors are eliminated, analgesics or calcium channel blockers should be administered for headache relief. Notably, indomethacin and triptans must be avoided as they may precipitate RCVS [92]. Antiepileptic medications should be prescribed for symptomatic seizures but long-term use is generally unnecessary [92]. Glucocorticoids should also be avoided, since they can significantly worsen outcomes [92].

Nimodipine is the most commonly used calcium channel antagonist, followed by verapamil and nicardipine. The recommended dose of oral nimodipine is 30–60 mg every 4 h, with treatment duration ranging from a few days to 4–8 weeks [92].

Actually, the benefit of calcium channel blockers on cerebral vasoconstriction or stroke severity remains unclear [93]. A prospective observational study conducted in 2019 demonstrated that early administration of nimodipine (6–13 days post-onset) was independently linked to a shorter clinical course [94]. Nimodipine effectively prevented recurrent thunderclap headaches (TCHs), with few complications reported. The authors suggested that nimodipine could stabilize vasoconstriction and subsequent vasodilation in distal small arteries, potentially halting the centripetal spread of vasoconstriction and reducing the likelihood of complications.

Some case reports have shown clinical and angiographic benefit with intra-arterial administration of vasodilators. However, this intra-arterial approach carries a significant risk of complication including ischemic stroke, arterial perforation or dissection, and reperfusion injury including brain hemorrhages and edema [95]. Therefore, this strategy may be reserved for patients who develop severe vasospasm, ischemic or hemorrhagic manifestations, and associated conditions like PRES [92,96].

The intrathecal administration of calcium channel blockers has been proposed in order to minimize the risk of diffuse vasodilation and the subsequent oxygen compromise to end organs, which occur with systemic administration of these drugs [97].

Other treatments used in RCVS whose benefit is still unproven include intravenous magnesium [92], which has been mostly prescribed in postpartum cases, intra-arterial milrinone [98] and balloon angioplasty [99]. In particular, milrinone’s efficacy is likely related to its anti-inflammatory and vasodilatory effect as a phosphodiesterase type 3 inhibitor [100].

A few studies have explored the possible effects of prostacyclin on vasospasms secondary to SAH. These studies observed a markedly lower incidence of vasospasm and stroke in patients receiving endovenous prostacyclin (1 ng/kg/min) compared to the placebo group, along with a decrease in cerebral blood flow velocity (indicating a reduction in vasospasm) within 72 h after the start of prostacyclin infusion [101]. However, these results did not reach statistical significance.

Despite the high risk of ischemic stroke, there is no indication for antiplatelet agents or anticoagulants [12].

Furthermore, in cases of severe vasoconstriction, hypotension should be avoided. Given the sympathomimetic action of infused drugs in a hypotensive state, maintaining stable blood pressure through adequate hydration is preferred [12].

## 10. Conclusions

RCVS is a unifying term used to describe a group of disorders characterized by thunderclap headaches, which may or may not be associated with neurological complications, and reversible centripetal narrowing and dilatation of cerebral arteries. It exhibits a dynamic course, and despite its reversibility, clinical presentation can be severe and its complications dramatic. The pathophysiology remains unknown, necessitating further studies not only to understand its enigmatic origins but also to identify effective treatments.

## Figures and Tables

**Table 1:** Diagnostic criteria of headaches attributed to reversible cerebral vasoconstriction syndrome according to Headache Classification Committee of the International Headache Society (IHS).

Acute Headache Attributed to Reversible Cerebral Vasoconstriction Syndrome	Acute Headache Probably Attributed to Reversible Cerebral Vasoconstriction Syndrome
A. Any new headache fulfilling criterion C	A. Any new headache fulfilling criterion C
B. Reversible cerebral vasoconstriction syndrome (RCVS) has been diagnosed	B. Reversible cerebral vasoconstriction syndrome (RCVS) is suspected, but cerebral angiography is normal
C. Evidence of causation demonstrated by either or both of the following: 1. Headache, with or without focal deficits and/or seizures, has led to angiography (with “string of beads” appearance) and diagnosis of RCVS 2. Headache has one or more of the following characteristics: a. Thunderclap onset b. Triggered by sexual activity, exertion, the Valsalva maneuver, emotion, bathing and/or showering c. Present or recurrent during ≤1 month after onset, with no new significant headache after >1 month	C. Probability of causation demonstrated by all of the following: 1. At least two headaches within 1 month, with all three of the following characteristics: a. Thunderclap onset, and peaking in <1 min b. Severe intensity c. Lasting ≥ 5 min 2. At least one thunderclap headache has been triggered by one of the following: a. Sexual activity (just before or at orgasm) b. Exertion c. Valsalva-like maneuver d. Emotion e. Bathing and/or showering f. Bending 3. No new thunderclap or other significant headache occurs >1 month after onset
D. Either of the following: 1. Headache has resolved within 3 months of onset 2. Headache has not yet resolved but 3 months from onset have not yet passed	D. Either of the following: 1. Headache has resolved within 3 months of its onset 2. Headache has not yet resolved but 3 months from its onset have not yet passed
E. Not better accounted for by another ICHD-3 diagnosis	E. Not better accounted for by another ICHD-3 diagnosis

RCVS: reversible cerebral vasoconstriction syndrome. ICHD-3: International Classification of Headache Disorders—3rd edition.

**Table 2:** Hypothetical precipitating factors/triggers causing RCVS.

Various Precipitating Factors/Conditions and Triggers Causing RCVS
Drugs: selective serotonin (and noradrenaline) reuptake inhibitors, cyclophosphamide, fingolimod, tacrolimus, erythropoetine, intravenous immune globuline, red blood cell transfusion triptans, ergotamine, pseudoephedrine, cocaine, amphetamine derivatives, ecstasy, lysergic acid diethylamide, tetrahydrocannabinol, prednisolone and oral contraceptive pills
Early and late pregnancy, pre-eclampsia and (postpartum) eclampsia
Tumors: pheochromocytoma and neuroendocrine tumor (e.g., bronchial carcinoid)
Traumatic brain injury and neurosurgical procedures
Porphyria
Vascular conditions: post-carotid endarterectomy, unruptured cerebral aneurysm, spinal subdural hematoma and COVID-19
Triggers: laughing, coughing, bathing, Valsalva maneuver, exertion, emotion and sexual activity

RCVS: reversible cerebral vasoconstriction syndrome.

**Table 3:** Different causes of narrowing of cerebral arteries and thunderclap-like headache.

Variable	RCVS	PACNS	SAH-Induced Vasospasm
CSF findings	Usually normal or mild increase in protein levels and mild pleocytosis	Abnormal in up to 90%, moderate pleocytosis, elevated levels of proteins, presence of oligoclonal bands and intrathecal IgG synthesis	Xanthochromia, elevated red blood cell count
Parenchymal and vascular brain imaging findings	Normal MRI or presence of watershed ischemic stroke, parieto-occipital stressed vasogenic edema (PRES), parenchymal (lobar and deep) hemorrhages or cSAH Widespread, symmetrical tapering reversible vasoconstriction and vasodilation of intracranial vessels	Acute/subacute/ chronic disseminated ischemic lesions in gray and white matter, hemorrhagic lesions with mass effect and possible white matter lesions Irregular, eccentric, notched with distal cutoffs, irreversible Multiple, irreversible intracranial stenoses Meningeal and vessel wall enhancement	Blood in the sylvian fissure and the basal cisterna Possible ruptured aneurysm or arteriovenous malformation; reversal segmental narrowing occurs after 4–14 days

RCVS: reversible cerebral vasoconstriction syndrome. PACNS: primary angiitis of the central nervous system. SAH: subarachnoid hemorrhage. PRES: posterior reversible encephalopathy syndrome. cSAH: convexity subarachnoid hemorrhage.

**Table 4:** RCVS_2_ score.

Criteria	Value
Recurrent or single TCH	Present 5 Absent 0
Involvement of intracranial carotid artery	Affected −2 Not affected 0
Presence of a vasoconstrictive trigger	Present 3 Absent 0
Sex	Female 1 Male 0
Presence of subarachnoid hemorrhage	Present 1 Absent 0

TCH: thunderclap headache.
